# MoSe_2_-GO/rGO Composite Catalyst for Hydrogen Evolution Reaction

**DOI:** 10.3390/polym10121309

**Published:** 2018-11-27

**Authors:** Wenwu Guo, Quyet Van Le, Amirhossein Hasani, Tae Hyung Lee, Ho Won Jang, Zhengtang Luo, Soo Young Kim

**Affiliations:** 1 School of Chemical Engineering and Materials Science, Chung-Ang University, 84 Heukseok-ro, Dongjak-gu, Seoul 06974, Korea; guowenwu95@gmail.com (W.G.); quyetbk88@gmail.com (Q.V.L.); amirhossein.hasani88@gmail.com (A.H.); 2 Institute of Research and Development, Duy Tan University, Da Nang 550000, Vietnam; 3 Department of Materials Science and Engineering, Research Institute of Advanced Materials, Seoul National University, Seoul 08826, Korea; sunshinety@snu.ac.kr (T.H.L.); hwjang@snu.ac.kr (H.W.J.); 4 Department of Chemical and Biomolecular Engineering, The University of Hong Kong Science and Technology, Clear Water Bay, Kowloon, Hong Kong

**Keywords:** transition metal dichalcogenides, electrocatalyst, hydrogen evolution reaction, molybdenum selenide composites

## Abstract

There has been considerable research to engineer composites of transition metal dichalcogenides with other materials to improve their catalytic performance. In this work, we present a modified solution-processed method for the formation of molybdenum selenide (MoSe_2_) nanosheets and a facile method of structuring composites with graphene oxide (GO) or reduced graphene oxide (rGO) at different ratios to prevent aggregation of the MoSe_2_ nanosheets and hence improve their electrocatalytic hydrogen evolution reaction performance. The prepared GO, rGO, and MoSe_2_ nanosheets were characterized by X-ray powder diffraction, Raman spectroscopy, X-ray photoelectron spectroscopy, transmission electron microscopy, energy-dispersive X-ray spectroscopy, and scanning electron microscopy. The electrocatalytic performance results showed that the pure MoSe_2_ nanosheets exhibited a somewhat high Tafel slope of 80 mV/dec, whereas the MoSe_2_-GO and MoSe_2_-rGO composites showed lower Tafel slopes of 57 and 67 mV/dec at ratios of 6:4 and 4:6, respectively. We attribute the improved catalytic effects to the better contact and faster carrier transfer between the edge of MoSe_2_ and the electrode due to the addition of GO or rGO.

## 1. Introduction

Hydrogen has received significant attention as one of the most environmentally friendly and efficient energy sources to replace current traditional fossil fuels [1,2]. Electrocatalytic H_2_ production from water splitting is a widely studied H_2_ production technology [3,4,5,6]. Platinum is the best-known catalyst for the electrocatalytic hydrogen evolution reaction (HER) [7,8,9]. However, its large-scale application is limited by its scarcity and high cost. Therefore, much research has been done to find an earth-abundant, efficient catalyst material with an HER with performance comparable to that of Pt. Recently, two dimensional transitional metal dichalcogenides (2D-TMDs) have been intensively investigated and employed in various applications such as solar cells, light emitting diodes, gas sensors, and HER [10,11,12,13,14,15]. Especially, the performance of 2D-TMDs as catalysts for HER approaching Pt has been demonstrated by several groups, revealing their potential in practical applications [16,17,18,19,20,21,22,23].

MoSe_2_ is a typical TMD material with layered structure, in which one layer of Mo atoms is sandwiched between two layers of Se atoms, and the layers are bound by weak van der Waals interaction [24,25,26]. The excellent catalytic activity of MoSe_2_ has been found to arise from the unsaturated Se atoms on the edges rather than the basal planes, which are electrochemically inert toward the HER; this has been confirmed by both theoretical calculations and experimental investigation in previous research [5,26,27,28,29]. Therefore, intense efforts have been made to synthesize MoSe_2_ or other metal dichalcogenide nanostructures with a high density of active edge sites [28,29,30,31]. Another problem that limits the catalytic activity of MoSe_2_ is its low conductivity, which limits electron transport between the electrode and electrocatalyst. To solve this conductivity problem and improve the electrocatalytic performance of MoSe_2_, combining MoSe_2_ nanosheets with other highly conductive materials such as porous carbon [32], reduced graphene oxide (rGO) [33], and metal oxides [34] is thought to be an effective strategy and has been sufficiently demonstrated in previous research. However, this method generally involves in situ growth of nanosheets on a conductive carbon substrate, so it is hard to control the size of the nanostructure.

In this study, we successfully prepared MoSe_2_ nanosheets using a modified colloidal synthesis method. The as-synthesized MoSe_2_ was prone to aggregation and showed unsatisfactory HER performance, with a somewhat high Tafel slope of 80 mV/dec. However, after it was mixed with GO or rGO at different ratios, we discovered that the aggregation problem was solved, and the composites tended to be uniformly distributed on the substrate, which was confirmed by field emission scanning electron microscopy (FE-SEM). Owing to this uniformity, along with the conductivity of rGO, the HER performance of the composites was better than that of the bare MoSe_2_ nanosheets. In addition, the resistance between the catalyst material and electrode was decreased, as calculated by electrochemical impedance spectroscopy (EIS) analysis. We identified this as one of the reasons for the catalytic performance improvement of the MoSe_2_ composites.

## 2. Materials and Methods 

### 2.1. Materials

All of the chemicals used in this work, including graphite powder, sodium nitrate (NaNO_3_), potassium permanganate (KMnO_4_), hydrogen peroxide (H_2_O_2_, 30%), l-ascorbic acid (L-AA, Sigma-Aldrich, Seoul, Korea), molybdenum hexacarbonyl (Mo(CO)_6_, Sigma-Aldrich), selenium (99.999%, Sigma-Aldrich), oleic acid (OA, 85%, Fluka, Mexico City, Mexico), oleylamine (OAm, 80–90%, Acros Organics, Geel, Belgium), and dodecanethiol were of analytical grade and used as received without further purification.

### 2.2. Preparation of GO-MoSe_2_ and rGO-MoSe_2_ Composites

#### 2.2.1. Synthesis of GO and rGO

GO was prepared using natural graphite powder by a modified Hummers method [35], and rGO was prepared following a previously reported method [36]. Briefly, 0.5 g of graphite powder and 0.5 g of NaNO_3_ were placed in a 250 mL flask containing 23 mL of concentrated H_2_SO_4_ and 3 g of KMnO_4_. The mixture was kept in an ice bath under strong stirring for 2 h. Next, the temperature of the reaction mixture was raised to 95 °C and held there for another 1 h. After the reaction was complete, the suspension was treated with H_2_O_2_ (10 mL, 30%) to remove the unreacted KMnO_4_ and then washed with water (55 mL); this was followed by dispersion in dimethylformamide (DMF) and sonication to obtain a GO/DMF solution. To obtain rGO nanosheets, 50 mg of L-AA was added to 50 mL of an aqueous dispersion of GO under vigorous stirring. GO was reduced for 48 h to remove most of the oxygen functionalities.

#### 2.2.2. Synthesis of MoSe_2_ Nanosheets 

MoSe_2_ nanosheets were prepared via a solution-processed colloidal method with a few important modifications to a previously reported method [26]. The synthesis was carried out using a three-neck flask under continuous N_2_ atmosphere in a heating mantle. In a typical procedure, 1.2 mmol Se powder was added to a mixture of OAm and dodecanethiol (9:1 vol %) in a three-necked flask. Then, the mixed solution was kept at 120 °C for 10 min under vigorous magnetic stirring to remove any water and oxygen; it was then heated to 200 °C for 1 h to produce a clear, light-yellow, highly active Se precursor solution. A Mo precursor solution was prepared by dissolving 0.6 mmol Mo(CO)_6_ in 9 mL of OA and 12 mL of acetone; it was then injected into the flask using a syringe at 2 drops/s after the Se solution was cooled to approximately 150 °C. Next, the mixtures were heated to 200 °C again and held for 30 min; they were then held at 250 °C for 30 min before being cooled rapidly by removal of the flask from the heating mantle. Subsequently, a large amount of hexane was added to the mixture, and the products were separated by centrifugation and washed repeatedly with hexane and ethanol. To totally remove the organic molecules, the products were further treated by dissolution in 30 mL of acetic acid, and the solution kept at 85 °C for 12 h with stirring. The products were washed with alcohol and centrifuged three times; finally, they were dried in a vacuum oven at 50 °C for further characterization.

#### 2.2.3. Synthesis of MoSe_2_-GO and MoSe_2_-rGO Composites

GO, rGO, and MoSe_2_ nanosheet powders were dissolved separately in DMF at a concentration of 1 mg/mL. Then, the GO and rGO solutions were each mixed separately with the MoSe_2_ nanosheet solution at volume ratios of 10:0 (no MoSe_2_ nanosheets), 8:2, 6:4, 4:6, 2:8, and 0:10 (only MoSe_2_ nanosheets). The mixtures were sonicated for approximately 1 h to ensure complete contact.

### 2.3. Material Characterization

The X-ray diffraction (XRD) patterns of the GO, rGO, and MoSe_2_ nanosheets were recorded on a powder X-ray diffractometer (Bruker New D8-Advance, Seoul, Korea) using Cu Kα radiation (*λ* = 0.154 nm). FE-SEM (Zeiss 300 VP, Seoul, Korea) images were obtained at an acceleration voltage of 10 kV to study the morphology of MoSe_2_ and the composites. Transmission electron microscopy (TEM, JEOL, Tokyo, Japan), along with energy-dispersive X-ray spectroscopy (EDX, JEOL, Tokyo, Japan) mapping, was performed using a JEOL (Tokyo, Japan) instrument to determine the size of the MoSe_2_ nanosheets and conduct elemental analysis. X-ray photoelectron spectroscopy (XPS, Thermo Fisher, K-Alpha, Seoul, Korea) was performed under a vacuum exceeding 1 × 10^−5^ mbar using Mg Kα radiation (1250 eV) and a constant pass energy of 40 eV to verify the presence of Mo and Se. Raman spectra (Horiba, Kyoto, Japan) were obtained at an excitation wavelength of 514 nm.

### 2.4. Electrochemical Measurements

HER measurements (Ivium Technologies, Nstat, Seoul, Korea) were made using a three-electrode system with a saturated calomel reference electrode, graphite rod counter electrode, and glassy carbon working electrode 3 mm in diameter in a 0.5 M H_2_SO_4_ standard electrolyte solution. The MoSe_2_ nanosheets and the composites dispersed in DMF were sonicated for 10 min before use. Then, 5 µL of catalyst ink was loaded onto the glassy carbon electrodes by drop casting and dried, followed by dropwise addition of 5 µL of Nafion solution (5 wt %) and further drying. Linear sweep voltammetry (LSV) curves were obtained at a sweep rate of 5 mV/s from 0.2 to 1.0 V versus reversible hydrogen electrode (RHE). EIS was performed by applying a constant potential of 0.27 V versus RHE in the frequency range of 100 kHz to 0.1 Hz with an amplitude of 10 mV. 

## 3. Results and Discussion

### 3.1. Synthesis of MoSe_2_ Nanosheets

The MoSe_2_ nanosheets were synthesized by injecting the Mo precursor, which was prepared by dissolving Mo(CO)_6_ in OAm and acetone, into a colloidal solution containing a high-activity Se source. Elemental Se has limited solubility in most solvents, so it has low reactivity for many reactions. Dodecanethiol can reportedly facilitate the dissolution of Se powder in OAm and lower the surface energy of the basal edges to prevent overgrowth of the basal plane [30]. During preparation of the Se precursor solution, the Se powder first dissolved completely in OAm and formed a dark red solution, which then gradually became transparent and light yellow, and was considered to be the highly active Se precursor solution [26,37]. Instead of cooling the Se precursor solution and then injecting it into the reaction flask after decomposition of Mo(CO)_6_, which was generally the procedure in previous research [26,30], the dissolved Mo(CO)_6_ was slowly injected into the Se solution while it retained its high activity. It is reported that without the Se precursor, the Mo(CO)_6_ precursor would first thermally decompose into Mo atoms and CO (Mo(CO)_6_ → Mo + 6CO), and then the Mo atoms would be oxidized to Mo_2_C by CO (Mo + 3CO → 1/2Mo_2_C + 3/2CO_2 _+ C) [38]. However, in our work, when the Mo(CO)_6_ precursor was slowly injected into the reaction system in the presence of the high-activity Se precursor, the metallic Mo would react with Se directly and quickly, as MoSe_2_ is thermodynamically more stable than molybdenum carbide [39]. This modification in our method was expected to ensure direct, rapid formation of MoSe_2_ nanosheets. Further, the mixed solvent of OAm and OA were expected to facilitate the formation of MoSe_2_ with porous nanostructure, which can increase the exposure of active edges.

### 3.2. Material Characterization

#### 3.2.1. Characterization of GO and rGO

**Figure 1:**
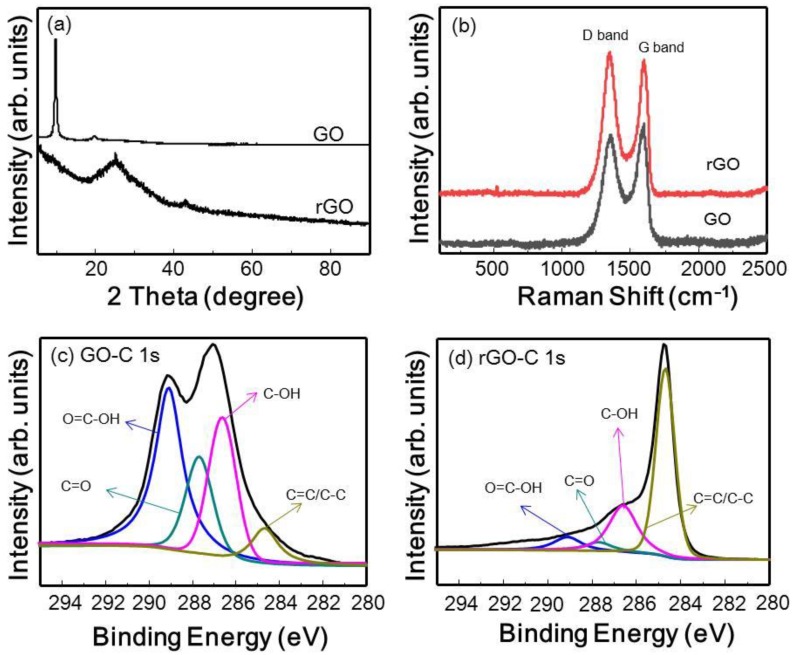
Characterization of graphene oxide (GO) and reduced graphene oxide (rGO): (**a**) X-ray diffraction (XRD) patterns; (**b**) Raman spectra; and (**c**) C 1s XPS spectra of GO and (**d**) C 1s XPS spectra of rGO.

The GO and rGO were characterized using XRD, XPS, and Raman spectroscopy. Figure 1a shows the XRD patterns of the GO and rGO after reduction. A sharp diffraction peak clearly appears around 11° for the GO sheets; after GO was reduced to rGO, this peak disappeared, and instead a new broad diffraction peak appeared at 24°. This result confirms successful reduction of GO and is consistent with previously reported results [36]. In addition, the laser Raman spectra of GO and rGO are shown in Figure 1b; the D and G bands are centered at 1354 and 1598 cm^−1^ for GO and 1351 and 1602 cm^−1^ for rGO, respectively. The variation in the relative intensities of the G band (the E_2g_ mode of sp^2^ carbon atoms) and D band (the symmetric A_1g_ mode) in the Raman spectra of GO during reduction usually reveals a change in the electronic conjugation state [37]. In GO, the G band appeared slightly more intense than the D band; however, after reduction to rGO, the D band was more intense than that of GO, and the D/G intensity ratio increased significantly, indicating reduction of GO. In addition, the XPS spectra of GO and rGO further proved that most of the oxygen functionalities were removed after 48 h of reduction. As the C 1s XPS spectra of GO in Figure 1c show, the four characteristic peaks located at binding energies of 284. 5, 286.5, 287.7, and 288.7 eV correspond to the C=C/C–C, C–O, C=O, and O=C–H groups, respectively. After reduction, the intensity of the C–O, C=O, and O=C–H peaks, which indicate the existence of oxygen-containing groups, decreased greatly, as shown in Figure 1d.

#### 3.2.2. Characterization of MoSe_2_

**Figure 2:**
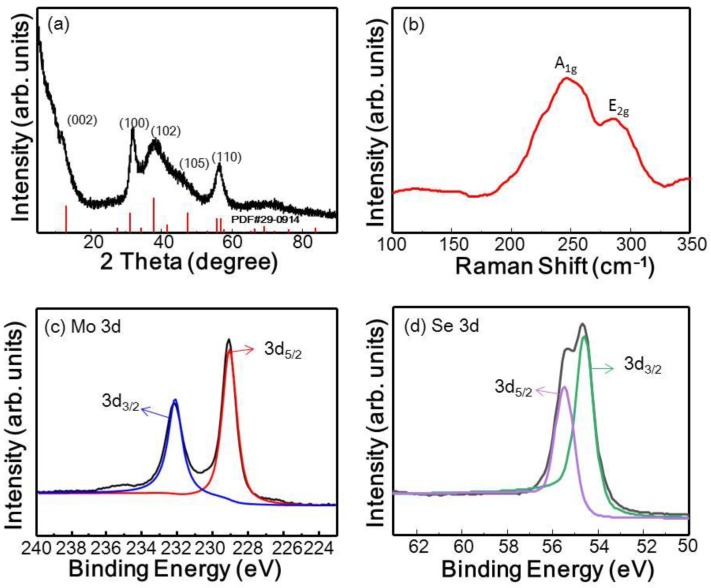
Compositional characterization of MoSe_2_ nanosheets: (**a**) XRD pattern; (**b**) Raman spectrum; and high-resolution (**c**) Mo 3d; and (**d**) Se 3d X-ray photoelectron spectroscopy (XPS) spectra.

The XRD patterns of the synthesized MoSe_2_ nanosheets are shown in Figure 2a. The characteristic peaks at 2θ = 13.60°, 31.52°, 37.72°, and 55.67° are assigned to the (002), (100), (103), and (110) crystal planes of hexagonal MoSe_2_, respectively. These results well matched the reference pattern of hexagonal 2H-MoSe_2_ (PDF card number 29-0914), indicating the high purity of the as-synthesized product obtained by our method. Further, the broad diffraction peaks revealed the nanoscale dimensions of the products [40]. Further insight into the structure of the MoSe_2_ nanosheets was obtained from the Raman spectrum, as shown in Figure 2b. It shows two pronounced peaks centered at 246 and 284 cm^−1^, which were assignable to the out-of-plane A_1g_ and in-plane E_2g_ vibration modes of MoSe_2_, respectively, and show a red shift compared to those of MoSe_2_ bulk materials [41]. The surface elemental composition and binding energy of the as-synthesized MoSe_2_ nanosheets were further investigated by XPS. As shown in Figure 2c and d, the two characteristic peaks at binding energies of 232.15 and 229.08 eV can be assigned to the Mo 3d_3/2_ and Mo 3d_5/2_ orbitals, respectively, indicating that Mo is in the +4 valence state. In addition, the characteristic peaks arising from the Se 3d_5/2_ and Se 3d_3/2_ orbitals are located at 55.38 and 54.68 eV and reveal the −2 oxidation state of Se. 

**Figure 3:**
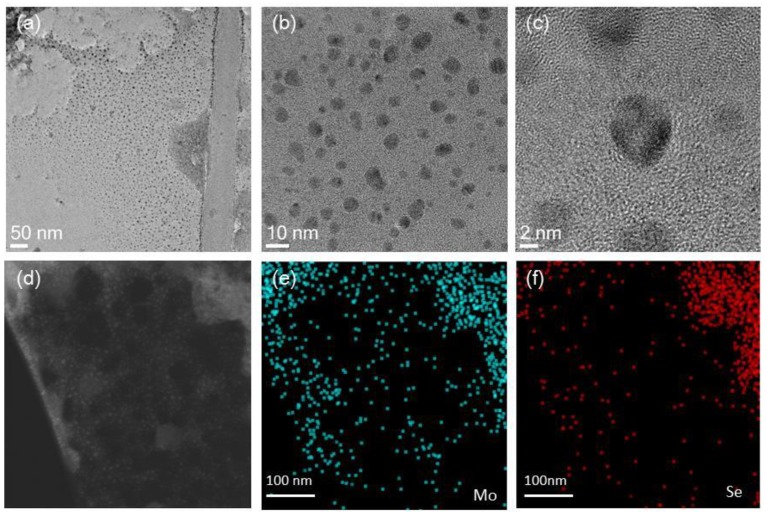
Microscopic characterization of MoSe_2_ nanosheets: transmission electron microscopy (TEM) images of MoSe_2_ nanosheets at (**a**) low; (**b**) medium; and (**c**) high magnification; (**d**) TEM image of MoSe_2_ nanosheet and corresponding elemental maps of (**e**) Mo and (**f**) Se.

Figure 3a–c show TEM images of the as-obtained MoSe_2_ nanosheets at different magnifications. All the particles had nanoscale dimensions, and the diameter of the MoSe_2_ nanosheets was approximately 2–10 nm, which is consistent with the small size of the products indicated by the XRD results. More details about the size distribution of as-obtained MoSe_2_ nanosheets are shown in Figure S1. Moreover, the EDX elemental mapping results shown in Figure 3e,f reveal the existence of Mo and Se and their homogeneous distribution throughout the ultrathin nanosheets.

#### 3.2.3. Characterization of MoSe_2_ Composites

**Figure 4:**
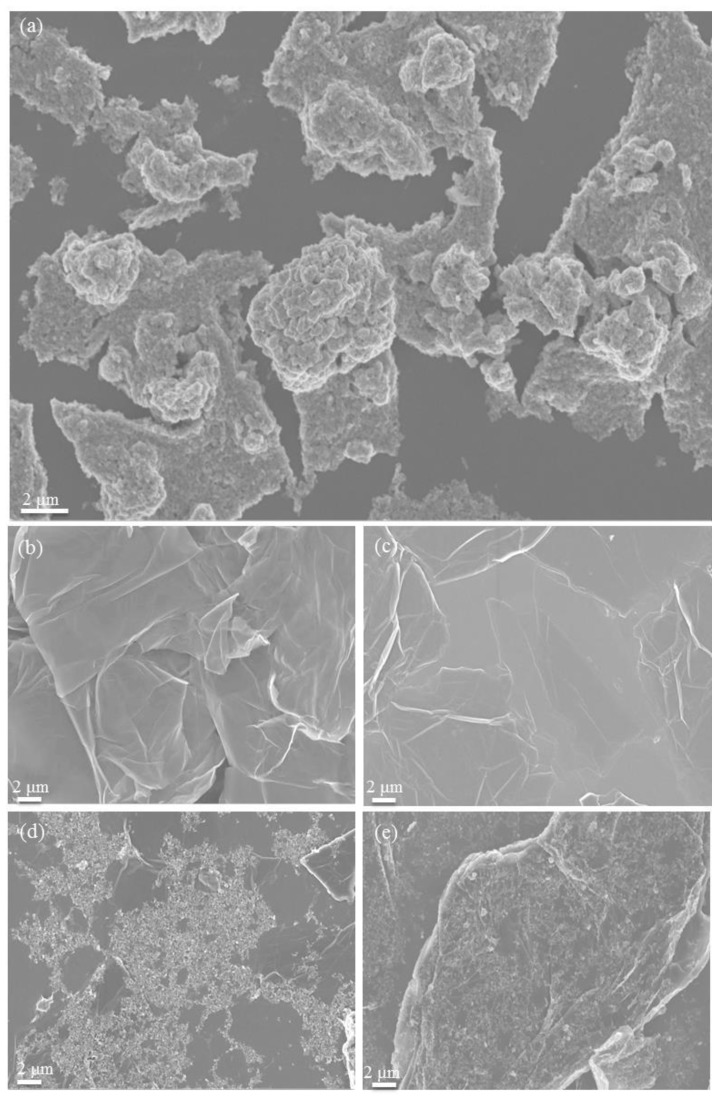
SEM images of (**a**) as-obtained MoSe_2_; (**b**) GO; (**c**) rGO; (**d**) GO-MoSe_2_-6:4; and (**e**) rGO-MoSe_2_-4:6.

FE-SEM measurement was conducted to investigate the morphological differences between the MoSe_2_ nanosheets before and after mixing with GO and rGO. Figure 4a–d shows FE-SEM images of pure MoSe_2_ nanosheets, GO, rGO, and the GO-MoSe_2_-6:4 and rGO-MoSe_2_-4:6 composites dried on a Si substrate. Images of the composites with other ratios are shown in Figures S2 and S3 (Supporting Information). The results showed that the pure MoSe_2_ nanosheets were prone to aggregation and formation of large particles. However, after mixing with GO and rGO, the composites showed a uniform distribution on the Si surface. Compared to other MoSe_2_ composites demonstrated by previous works, our mixing process is expected to be advantageous in terms of its efficiency and its ease of operability and scalability.

### 3.3. Electrocatalytic Properties

**Figure 5:**
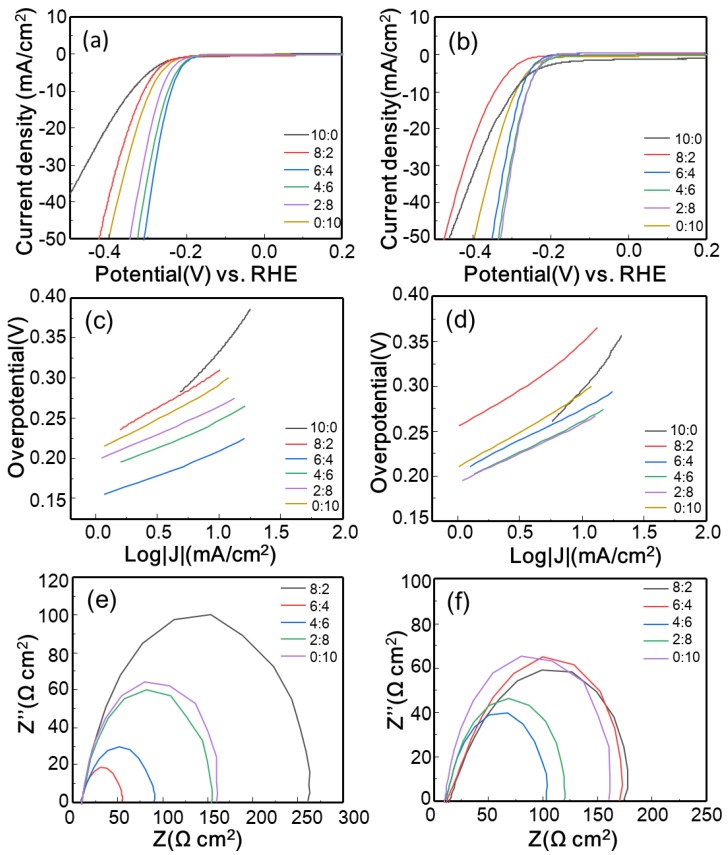
Electrochemical measurements: polarization curves of (**a**) GO-MoSe_2_ composites; (**b**) rGO-MoSe_2_ composites; and (**c**) and (**d**) corresponding Tafel plots; electrochemical impedance spectra of (**e**) GO-MoSe_2_ and (**f**) rGO-MoSe_2_.

The HER electrocatalytic activities of MoSe_2_ mixed with GO or rGO at different ratios were evaluated using LSV. As shown in Figure 5a,b, pristine MoSe_2_ nanosheets (ratio of 0:10) exhibited a somewhat high overpotential of approximately 210 mV at a cathode current density of 1 mA/cm^2^ (*η*_1_), but the current density was substantially enhanced after GO or rGO was added. The *η*_1_ values of most of the GO-MoSe_2_ and rGO-MoSe_2_ composites (the sample with a ratio of 10:0 is not included among the composites here or in the following discussion) were lower than that of the pristine MoSe_2_. In particular, the GO-MoSe_2_-6:4 (GO-MoSe_2_ with a ratio of 6:4) and rGO-MoSe_2_-4:6 samples exhibited the lowest *η*_1_ values of 180 and 194 mV, respectively. To further investigate their HER catalytic activity, the overpotential at a cathode current density of 10 mA/cm^2 ^(*η*_10_), which is usually regarded as an indicator of HER performance [8], was also recorded for the GO-MoSe_2_ and rGO-MoSe_2_ composites. Compared to the pure MoSe_2_ (295 mV versus RHE), the *η*_10_ values of GO-MoSe_2_-6:4 and rGO-MoSe_2_-2:8 decreased to 238 and 256 mV versus RHE, respectively. The *η*_1_ and *η*_10_ values of the other samples are summarized in Table 1. Tafel analysis was also performed to evaluate the HER performance of the catalyst. The results are derived from the polarization curve and fitted by the Tafel equation: *η* = *b*log*j* + *a*, where *η*, *b*, and *j* represent the overpotential, Tafel slope, and current density, respectively [29,42]. As shown in Figure 5c,d, the GO-MoSe_2_ and rGO-MoSe_2_ composites all exhibited smaller Tafel slope than the pure MoSe_2_ nanosheets (80 mV/dec), except for the one with a ratio of 8:2 (89 mV/dec for GO-MoSe_2_ and 88 mV/dec for rGO-MoSe_2_). Especially, the Tafel slopes were reduced to 57 and 66 mV/dec for GO-MoSe_2_-6:4 and rGO-MoSe_2_-4:6, respectively. The results are in agreement with the above LSV analysis and suggest that GO and rGO can improve the HER performance of MoSe_2_ nanosheets even when they are added by simple mixing. Additionally, both pristine MoSe_2_ and best-performed MoSe_2_ composites outperformed most of the previously reported MoSe_2_ or MoSe_2_-based HER electrocatalysts (detailed comparisons are provided in Table S1), which indicates the superiority of our method. 

EIS was performed to study the reactions at the electrode/solution interface and the electron transfer kinetics in the HER process [42]. The Nyquist plots obtained from the impedance measurement were fitted with an equivalent circuit, as shown in Figure 5e,f, to determine the charge transfer resistance (*R*_ct_). Table 1 summarizes the *R*_ct_ values of all the samples. All of the MoSe_2_ composites have smaller *R*_ct_ values than pure MoSe_2_, except for the composites with a ratio of 8:2. Among them, GO-MoSe_2_-6:4 and rGO-MoSe_2_-4:6 showed the smallest *R*_ct_ values of 35.4 and 79.1 Ω cm^2^, respectively. *R*_ct_ is related to the electrocatalytic kinetics at the interface between MoSe_2_ and the electrolyte. Further, a lower *R*_ct_ indicates faster electron transfer at the interfaces [43]. Therefore, it can be assumed that the conductive GO and rGO can promote electron transfer between MoSe_2_ and the electrolyte and hence improve the HER performance.

**Figure 6:**
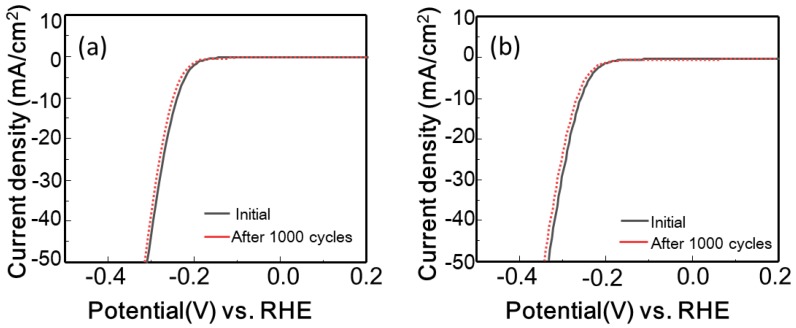
Stability tests: polarization curves of (**a**) GO-MoSe_2_-6:4 and (**b**) rGO-MoSe_2_-4:6 initially and after 1000 cycles.

To assess the stability of the MoSe_2_ composites exhibiting the best performance (GO-MoSe_2_-6:4 and rGO-MoSe_2_-4:6), cyclic voltammetry tests between −0.4 and 0.2 V versus RHE were conducted at 50 mV/s for 1000 cycles. As shown in Figure 6a,b, the polarization curves of the MoSe_2_ composites before and after 1000 cycles almost overlap, indicating their stability in the HER.

## 4. Conclusions

In summary, we successfully synthesized MoSe_2_ nanosheets via a modified solution-processed method. GO and rGO were obtained by a previously reported method and applied to improve the HER performance of the as-synthesized MoSe_2_ nanosheets. XRD, XPS, Raman spectroscopy, FE-SEM, and TEM were used to characterize the structure and morphology of the nanosheets and composites. The results revealed that the MoSe_2_ nanosheets were in the 2H phase and had small sizes of approximately 2–10 nm, and the GO sheets were effectively reduced to rGO sheets. FE-SEM images suggested that the as-obtained MoSe_2_ nanosheets were prone to aggregation into large particles, but the aggregation issue was ameliorated in the GO-MoSe_2_ and rGO-MoSe_2_ composites. Moreover, electrochemical measurements further verified that the HER performance of the composites was better than that of the pristine MoSe_2_ nanosheets, and the GO-MoSe_2_-6:4 and rGO-MoSe_2_-4:6 composites showed low Tafel slopes of 57 and 66 mV/dec, respectively. This result suggests that the HER activity of MoSe_2_ nanosheets can be improved by adding GO or rGO at an appropriate ratio.

## Figures and Tables

**Table 1:** Summary of *η*_1_, *η*_10_, Tafel slope, and *R*_ct_ of GO-MoSe_2_ and rGO-MoSe_2_, with different ratios.

			GO-MoSe_2_				rGO-MoSe_2_	
	*η*_1_ (mV)	*η*_10_ (mV)	Tafel Slope (mV/dec)	*R*_ct_ (Ω cm^2^)	*η*_1_ (mV)	*η*_10_ (mV)	Tafel Slope (mV/dec)	*R*_ct_ (Ω cm^2^)
10:0	−43	333	164	819	−304	295	166	476
8:2	210	293	89	176	255	349	88	148
6:4	180	238	57	35.4	202	274	70	134
4:6	190	248	63	54.7	194	261	66	79.1
2:8	195	264	67	118	194	256	67	89.6
0:10	210	295	80	134	210	295	80	134

## Supplementary Materials

The following are available online at http://www.mdpi.com/2073-4360/10/12/1309/s1: Figure S1. Size distribution of as-obtained MoSe2 nanosheets; Figure S2: SEM images of GO-MoSe_2_ with different ratios: (a) 8:2; (b) 6:4; (c) 4:6; and (d) 2:8; Figure S3: SEM images of rGO-MoSe_2_ with different ratios: (a) 8:2; (b) 6:4; (c) 4:6; and (d) 2:8; Table S1. Comparison of the hydrogen evolution reaction (HER) performance between previous works and our work.
