# Supplementary material for: MoSe2-GO/rGO Composite Catalyst for Hydrogen Evolution Reaction
Source: Polymers (Basel). 2018 Nov 27;10(12):1309. doi: 10.3390/polym10121309 (PMC6401761; doi:10.3390/polym10121309)
Supplement: Supplementary file 1 [file polymers-10-01309-s001.pdf]

Supplementary Materials: $MoSe_2$-GO/rGO Composite Catalyst for Hydrogen Evolution Reaction

Wenwu Guo, Quyet Van Le, Amirhossein Hasani, Tae Hyung Lee, Ho Won Jang, Zhengtang Luo and Soo Young Kim

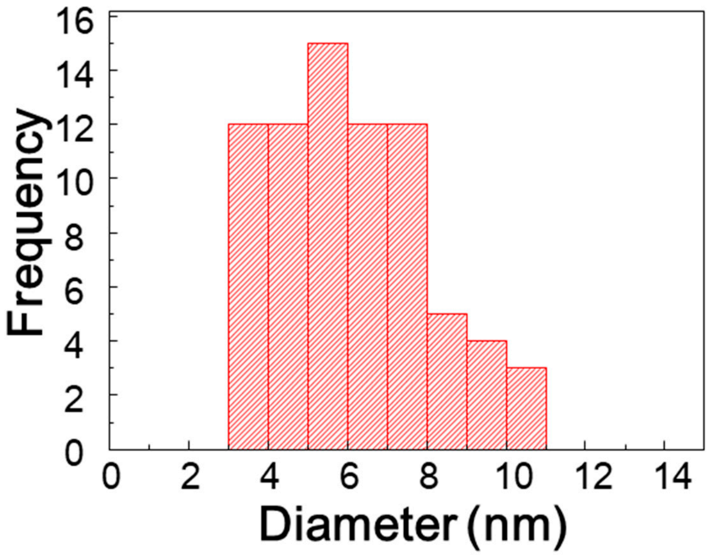

Figure S1. Size distribution of as-obtained $MoSe_2$ nanosheets.

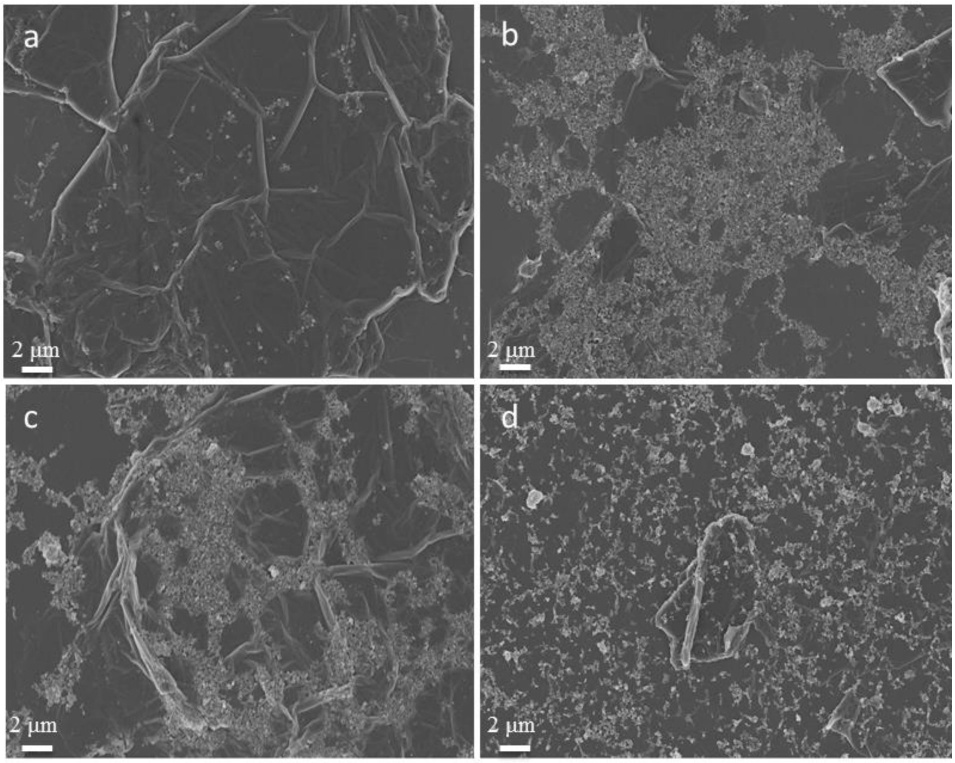

Figure S2. SEM images of GO-$MoSe_2$ with different ratios: (**a**) 8:2; (**b**) 6:4; (**c**) 4:6; and (**d**) 2:8.

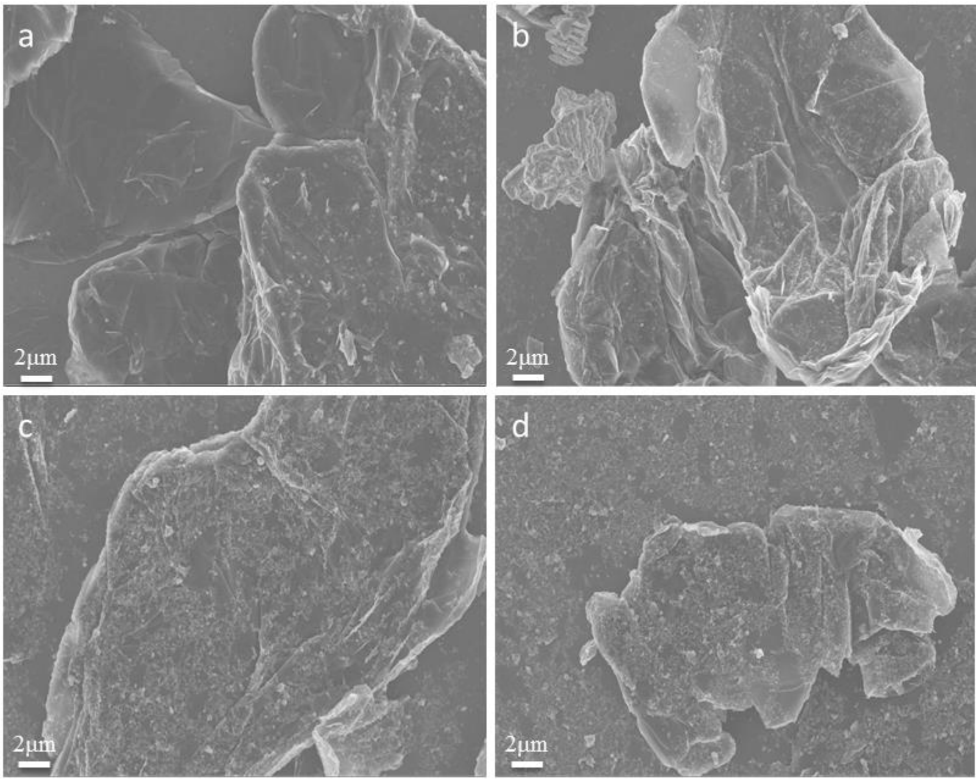

Figure S3. SEM images of rGO-$MoSe_2$ with different ratios: (**a**) 8:2; (**b**) 6:4; (**c**) 4:6; and (**d**) 2:8.

Table S1. Comparison of the hydrogen evolution reaction (HER) performance between previous works and our work.

Electrocatalyst	Synthesis Method	Tafel Slop (mV/dec)	Ref.
$MoSe_2$/RGO hybrid	hydrothermal	69	[1]
$MoSe_2$/carbon fiber	hydrothermal	70	[2]
$MoSe_2$/$CoSe_2$	hydrothermal	73	[3]
CNT@$MoSe_2$	Solvothermal	58	[4]
$MoSe_2$	Colloid method	102	[5]
S-doped $MoSe_2$	Colloid method	60	[5]
Mo-rich $MoSe_2$	Colloid method	98	[6]
$MoSe_2$/graphene	Colloid method	67	[7]
$MoSe_2$	Colloid method	89	[8]
$MoSe_2$	Colloid method	80	This work
$MoSe_2$/GO	Colloid method	57	This work
$MoSe_2$/rGO	Colloid method	67	This work

References

1. Tang, H.; Dou, K.; Kaun, C.-C.; Kuang, Q.; Yang, S. $MoSe_2$ nanosheets and their graphene hybrids: synthesis, characterization and hydrogen evolution reaction studies. *J. Mater. Chem. A* **2014**, *2*, 360–364.
2. Qu, B.; Yu, X.; Chen, Y.; Zhu, C.; Li, C.; Yin, Z.; Zhang, X. Ultrathin $MoSe_2$ nanosheets decorated on carbon fiber cloth as binder-free and high-performance electrocatalyst for hydrogen evolution. *ACS Appl. Mater. Interfaces* **2015**, *7*, 14170–14175.
3. Mu, C.; Qi, H.; Song, Y.; Liu, Z.; Ji, L.; Deng, J.; Liao, Y.; Scarpa, F. One-pot synthesis of Nanosheet-assembled hierarchical $MoSe_2$/$CoSe_2$ microcages for the enhanced performance of electrocatalytic hydrogen evolution. *RSC Adv.* **2016**, *6*, 23–30.
4. Huang, Y.; Lu, H.; Gu, H.; Fu, J.; Mo, S.; Wei, C.; Miao, Y.-E.; Liu, T. A CNT@$MoSe_2$ hybrid catalyst for efficient and stable hydrogen evolution. *Nanoscale* **2015**, *7*, 18595–18602.
5. Xu, C.; Peng, S.; Tan, C.; Ang, H.; Tan, H.; Zhang, H.; Yan, Q. Ultrathin S-doped $MoSe_2$ nanosheets for efficient hydrogen evolution. *J. Mater. Chem. A* **2014**, *2*, 5597–5601.

6. Zhou, X.; Jiang, J.; Ding, T.; Zhang, J.; Pan, B.; Zuo, J.; Yang, Q. Fast colloidal synthesis of scalable Mo-rich hierarchical ultrathin $MoSe_{2-x}$ nanosheets for high-performance hydrogen evolution. *Nanoscale* **2014**, *6*, 11046–11051.
7. Liu, Z.; Li, N.; Zhao, H.; Du, Y. Colloidally synthesized $MoSe_2$/graphene hybrid nanostructures as efficient electrocatalysts for hydrogen evolution. *J. Mater. Chem. A* **2015**, *3*, 19706–19710.
8. Guo, W.; Chen, Y.; Wang, L.; Xu, J.; Zeng, D.; Peng, D.-L. Colloidal synthesis of $MoSe_2$ nanonetworks and nanoflowers with efficient electrocatalytic hydrogen-evolution activity. *Electrochim. Acta* **2017**, *231*, 69–76.

© 2018 by the authors. Submitted for possible open access publication under the terms and conditions of the Creative Commons Attribution (CC BY) license (http://creativecommons.org/licenses/by/4.0/).
